# AMELX and ENAM Polymorphisms and Dental Caries

**DOI:** 10.1155/2022/8501179

**Published:** 2022-12-31

**Authors:** Mohammad Reza Khami, Sahar Asgari, Sara Valizadeh, Jafar Karami, Arezou Rezaei, Nima Rezaei

**Affiliations:** ^1^Research Center for Caries Prevention Dentistry Research, Institute Community Oral Health Department, School of Dentistry, Tehran University of Medical Sciences, Tehran, Iran; ^2^Department of Periodontology, School of Dentistry, Babol University of Medical Sciences, Babol, Iran; ^3^Department of Operative Dentistry, School of Dentistry, Tehran University of Medical Sciences, Tehran, Iran; ^4^Molecular and Medicine Research Center, Khomein University of Medical Sciences, Khomein, Iran; ^5^Research Center for Immunodeficiencies, Children's Medical Center, Tehran University of Medical Sciences, Tehran, Iran; ^6^Department of Immunology, School of Medicine, Tehran University of Medical Sciences, Tehran, Iran; ^7^Network of Immunity in Infection, Malignancy and Autoimmunity (NIIMA) Universal Scientific Education and Research Network (USERN), Tehran, Iran

## Abstract

**Introduction:**

The variety of the genetic factors playing role in development of dental caries calls for further research in this regard. The aim of the present study was to investigate the differences between caries-free adults and adults with dental caries in terms of polymorphism of caries-related genes (AMELX and ENAM).

**Methods:**

The present case-control study was performed on 81 adults aged 18–24 years, 41 caries free, and 40 with a DMFT ≥ 4. A questionnaire containing background and demographic information (such as age, gender, time and type of latest dental check-ups, parent's education, oral self-care, and the place of residence in the first 12 years of life) was completed by participants at the time of examination. The blood sample was taken from each participant in the EDTA tube, and PCR was performed. Gene diversity of AMELX and ENAM genes was compared between the two groups.

**Results:**

Regarding AMELX gene, in the caries-free group 33 (80.5%) and in the group with DMFT ≥ 4, 33 (82.5%) students had TT genotype, but this difference was insignificant. For ENAM gene, in the caries-free group 34 (82.9%) and in the group with DMFT ≥ 4, 39 (97.5%) students had TT genotype, but this difference was insignificant (*P* value = 0.048, CI 95%:0.02–1.27, and OR = 0.145).

**Conclusion:**

There was no relationship between TT and TC genotypes of single nucleotide polymorphism of *AMELX* and *ENAM* gene and susceptibility to dental caries, but with increasing sample size, there may be a relationship between SNP of *ENAM* gene and being caries free.

## 1. Introduction

Various types of genes interact at the beginning of tooth development, tooth formation and cell differentiation, including sonic hedgehog (SHH), patch (PTCH), WNT, and bone morphogenic protein 2,4 (BMP2 and BMP4). Other genes such as PAX9, BARX1, LEF1, DLX1, DLX2, MSX1, and MSX2 play a role in designing the tooth shape [1]. The first known markers for the tooth formation are LIM-homeobox genes including Lhx-6 and Lhx-7, which are induced by fibroblast growth factor-8 (FGF-8). Another gene that is expressed at the same stage in mesenchymal tissues and determines the location of tooth germ is paired box homeotic gene-9 (Pax-9) [2].

Four main categories of genes can potentially play a role in the development of dental caries: genes responsible for enamel evolution including AMELX, AMELY, ENAM, and AQP5; genes responsible for saliva production and synthesis including CA6 (carbonic anhydrase 6) and AQP5; genes responsible for immunological responses including LTF (encoding lactoferrin); and genes responsible for carbohydrate metabolism including ESRRB (oestrogen-related receptor *β*). Among them, the genes in the first category, specifically AMELX, ENAM (enamelin), AQP5, and ESRRB seem to be the most important ones [3–5].

Ameloblasts are enamel-forming cells that originate in ectoderm. Enamel is made in two steps. In the first step, enamel proteins, which are mainly amelogenin, are synthesized. The gene encoding amelogenin is AMELX [6]. Amelogenins are well organized for the formation of the hydroxyapatite prism and are essential for the elongation of the prisms during enamel development and for the production of normal enamel thickness. However, they are not necessary for the onset of enamel formation [7]. The ENAM gene also plays a key role in tooth enamel formation. It is thought that enamelin peptides composing around 5% of the enamel matrix contribute to the formation and elongation of enamel crystallites during tooth development [8]. ENAM is a member of the P/Q-rich calcium-binding phosphoprotein cluster genes.

Despite the prominence of tooth enamel thickness in the evolutionary studies of modern primates and hominids and genotypic studies, only one study elucidated the phenotypic effects of polymorphism [3]. Some genome-wide association studies in patients with dental caries from European Americans showed the role of types of ENAM genes in the formation of dental caries and aroused greater interest in the study of single nucleotide polymorphisms in the ENAM gene. Exogen 10 of the ENAM gene seems to plays a key role in causing dental caries. Previous studies have reported that ENAM genes including rs3796704 and rs7671281 may affect the microstructure of tooth enamel, which may lead to caries and may play a role in dental caries. Further research on the ENAM gene has led to the identification of nonsynonymous mononucleotide polymorphisms (rs7671281 and rs3796704) that may be associated with the formation of dental caries [4].

There are people in society who are still completely without caries or “caries free” even until old age. However, these people seem not to be different from other people in terms of known risk factors for dental caries, which increase the likelihood of some genetic resistance against dental caries. This hypothesis can be best considered in adults since the time passed form the eruption of tooth is a main factor in development of dental caries. However, most of the studies on the association of enamel coding genes and dental caries have been conducted among children. Moreover, to the best of our knowledge, those conducted in adults have not investigated caries-free individuals. Also, no study was found to examine the status of caries-related genes in the Iranian population.

Therefore, the aim of the present study was to investigate the differences between caries-free individuals and those with dental caries in terms of two genes associated with dental caries, i.e., AMELX and ENAM.

## 2. Materials and Methods

The present case-control study was conducted on students of Tehran University of Medical Sciences (18–24 years old). Upon their entrance to the university, TUMS students undergo a complete medical and dental examination. The record of this examination was obtained from the Students Health Center (SHC), and based on that, two groups of students were invited through phone call to participate in the study: caries-free group and those with a DMFT equal to or higher than the mean DMFT among Iranian 18-24-year olds, i.e., 4 [9]. Those who accepted were invited to TUMS School of Dentistry, and a specialist in restorative dentistry examined them and recorded their DMFT index. The dental examination was performed using probes, mirrors, and headlights, and DMFT was recorded based on the WHO criteria [10]. When the examination result differed from that of SHC, another student from the list was replaced. All invited students were offered to receive their dental treatment in the clinic of the school at subsidized cost and received individual oral health education. Those 81 students (41 in the caries-free group and 40 in the group with dental caries) who fulfilled the study criteria gave written informed consent to participate in the study, and this study was approved by the Research Ethics Committee of Tehran University of Medical Sciences (code: IR.TUMS.DENTISTRY.REC.1398.059). They received an oral health package of toothbrush and toothpaste as well.

The recruited students completed a questionnaire requesting information on age, gender, time and type of latest dental check-ups, parent's education, oral self-care, and the place of residence in the first 12 years of life. The latter was asked to determine the fluoride level of the drinking water of the participants' residence area during formation and eruption of permanent teeth, which was then determined by the researchers based on the results of national surveys on the fluoride level of drinking water. Based on these surveys, the students were placed in either low or very low category. Undergoing dental check up by a dentist and within the last year was defined as acceptable [11]. Recommended tooth brushing habit was defined as brushing more than once a day with regular or frequent use of fluoride toothpaste [12]. Consumption of snacks containing sugar was dichotomized to acceptable and nonacceptable (once a day or more) [11].

Three to four cc of blood was taken from each student in the EDTA tube. DNA was then extracted by the phenol chloroform method.

Table 1 shows primer design for two SNPs of AMELX and ENAM genes.

After performing PCR by ARMS PCR, gene diversity of AMELX and ENAM genes was compared between the two groups. PCR and genetic analysis were performed through the following steps: in the first step, amplification using 10 *μ*l of master mix, 12 *μ*l of sterile distilled water, 1 *μ*l of sample (20–100 ng DNA), and 1 *μ*l of each primer (pmol/10 *μ*L). The process was performed under the following conditions: initial denaturation at 95°C for 2 minutes, denaturation at 95°C for 30 seconds, annealing in the form of gradient at a temperature of 65, 64.5, 64, 63.5, 63, and 62.5°C (respectively for each pair of primers) for 30 seconds, and extension at 72°C for 30 seconds and 35 cycles. Presence and absence of specific band of PCR products became visible by gel document after gel electrophoresis using agarose gel.

### 2.1. Statistical Analysis

The data was analyzed by SPSS version 26 software. The Chi-square test and logistic regression model served for statistical analysis. The significance level was set at 0.05.

## 3. Results

Of the 81 students, 41 (50.6%) were male and 40 (49.4%) were female.  Table 2 shows the background information between the two study groups.

Regarding AMELX gene, of the 81 participants, 66 (81.5%) had TT genotype and 12 (14.8%) had TC genotype. Two (2.5%) of the participants had CC genotype, and the genotype of one student (1.2%) could not be determined. These three cases were excluded from further analysis. Based on the chi-square test to examine TT and TC genotypes, single nucleotide polymorphism of AMELX gene, in both the caries-free group and in the group with DMFT ≥ 4, 33 students had TT genotype (80.5% and 82.5%, respectively). The backward likelihood ratio binary logistic regression model was fitted to data related to the AMELX gene. Based on the model, those with a higher level of mother's education had a higher caries level (*P* = 0.004, OR = 0.169, and 95% CI: 0.051–0.558). Also, those who had their last check up during the last year (*P* = 0.036, OR = 3.922, and 95% CI: 1.096–14.043) and those who received higher water fluoride (*P* = 0.010, OR = 5.517, and 95% CI: 1.515–20.091) had a lower risk of caries. It should be noted that the AMELX gene genotype variable was not significantly correlated in any of the steps and was excluded from the model in step 4 (Table 3).

Regarding ENAM gene, of the 81 participants, 73 (90.1%) had TT genotype and 7 (8.6%) had TC genotype. The genotype of one case (1.2%) could not be determined, which were excluded from further analysis. Based on the chi-square test to examine TT and TC genotypes, single nucleotide polymorphism of ENAM gene, in the caries-free group 34 (82.9%), and in the group with DMFT ≥ 4, 39 (97.5%) students had TT genotype, and this difference was statistically significant (*P* value = 0.048, CI 95%: 0.02–1.27, and OR = 0.145). According to the backward likelihood ratio binary logistic regression model, female gender had a lower caries level (*P* = 0.046, OR = 4.010, and 95% CI: 1.022–15.733). Also, those with a higher level of mother's education had a higher caries level (*P* = 0.001, OR = 0.449, and 95% CI: 0.020–0.371), and those who had dental check up by dentist (*P* = 0.025, OR = 4.616, and 95% CI: 1.221–20.220), and those who received higher water fluoride (*P* = 0.010, OR = 6.201, and 95% CI: 1.556–24.711) had a lower risk of caries (Table 4).

The ENAM gene allele variable was not significantly related in any of the steps and was excluded from the model in the last step.

## 4. Discussion

The aim of the present study was to evaluate the differences between caries-free adults and adults with dental caries in terms of polymorphism of caries-related genes (AMELX and ENAM). Statistically significant association was found between TT and TC genotypes of single nucleotide polymorphism of ENAM gene in univariate analysis. However, the significant difference disappeared in multivariate analysis (regression model), showing that the difference probably has been the reflection of gender difference. That the female students were more likely to be caries free according to the regression model supports this hypothesis. On the other hand, according to the results, the relationship between single nucleotide polymorphisms of ENAM gene and the absence of dental caries was close to be significant and may become significant with increase in the sample size in further studies.

According to two separate regression models for AMELX and ENAM genes, there were statistically significant relationships between the maternal education, last dental check up, and water fluoride of the place where the person had spent most of his life before the age of 12 and the prevalence of caries, but there was no statistically significant relationship between dental check up and caries prevalence in AMELX gene model unlike ENAM gene. The AMELX gene and ENAM gene genotype variable showed no significant relationship in any of the model stages and were excluded from the model prior to the final stage of the models.

Sharifi et al. [13] in a meta-analysis study found that ENAM rs3796704 polymorphism, especially in the Caucasus ethnic group and in studies with caries-free individuals as a control group, had a higher risk of caries, but they found no association between polymorphisms LTF rs1126478, ENAM (rs1264848 and rs3796703), and AMELX (rs946252, rs17878486, and rs2106416) and susceptibility to dental caries. However, sensitivity analysis showed that AMELX rs17878486 polymorphism can be a risk factor for dental caries, but race and type of the control group are the factors influencing the relationship between AMELX rs17878486 polymorphism and the risk of dental caries. In the present study, there was no significant relationship between AMELX gene single nucleotide polymorphism and susceptibility to dental caries. Jeremias et al. [14] and Gerreth et al. [15] in a study showed a link between AMELX rs17878486 polymorphism and susceptibility to dental caries and found that the role of AMELX rs17878486 polymorphism in dental caries may be greater than others unlike the results of the present study. These differences can be attributed to differences in race and environmental conditions as well as implementation of different methods.

Bayram et al. [16] showed that ENAM polymorphisms may affect tooth enamel formation, and these effects may vary between deciduous and permanent teeth. Devang Divakar et al. [17] reported increased risk of ENAM rs1264848 and ENAM rs3796704 polymorphisms in a study of Saudi patients, while Gerreth et al. [15] found in a study of Polish children that the protective role of polymorphism ENAM rs1264848 is present in dental caries. Abbasoğlu et al. [18] also confirmed these findings in a study on Turkish children. In a study, Wang et al. [19] showed an increased risk of ENAM rs3796703 polymorphism in patients with dental caries compared to the group without caries. Borilova Linhartova et al. [20] in a study reported a significant difference in the frequency of minor alleles between the Polish and Czech populations and also confirmed the sensitivity analysis of the effect of ethnicity and type of the control group on the relationship between ENAM rs3796704 and the risk of dental caries. In the present study, there was no statistically significant relationship between the incidence of caries and single nucleotide polymorphism of ENAM gene.

In our study, the parent's education level was considered as an indicator of socio-economic status. The relationship between the level of education and occurrence of dental caries has been investigated in a number of studies. Siukosaari et al. [21] was unable to find a correlation between the education level and DMFT. Studies by Fox et al. [22], Locker and Leake [23], and Beck et al. [24] showed that the relative importance of education would diminish if other socio-economic factors such as the income level, recent dental visit, and age were considered. In the study of Khosravi et al. [25], health knowledge, attitude, and practice were higher in women than men and in educated people and in young people than illiterate and older people, respectively. In Demoura's study [26], low education and income as well as low frequency of brushing were identified as factors affecting caries activity. In the study by Zhu et al. [27], dental caries was also affected by urbanity, number of brushing times, and the brushing method and duration. In the present study, it was found that the incidence of caries increases with increasing maternal education, which also might be attributable to urbanity.

A body of sufficient evidence exists on the preventive effects of fluoride against dental caries. Fluoridation of the drinking water and of toothpastes is now considered the preferred mode of fluoride administration, appraised by the highest level of evidence [28]. In the present study, it was found that those who received higher water fluoride had a lower risk of caries. Moreover, that the incidence of dental caries was correlated with such upstream factors as maternal education and fluoride in drinking water rather than oral self-care and lifestyle factors is an indication of the importance of social determinants of health.

### 4.1. The Strengths and Weaknesses of the Study

The present study could not overcome some of the limitations because this study was conducted at the time of the outbreak of COVID 19 virus, and due to the pandemic of this virus, the desire to participate in this project was much lower, thus the sample size in this study was low and we could reach 81 students. Also, the method of gene sequencing was not performed in our study.

## 5. Conclusion

The findings of the present study suggested that there was no association between TT and TC genotypes of single nucleotide polymorphism of AMELX gene and ENAM gene with susceptibility to dental caries. The relationship between single nucleotide polymorphism of ENAM gene and the absence of caries was close to be significant. Thus, further research on the subject with larger sample size is suggested.

## Figures and Tables

**Table 1:** Primer design for two SNPs of AMELX and ENAM genes.

	rs17878486 (AMELX)	rs2609428 (ENAM)
Wildtype	AGAGAATAAACCTTCCCATGAACT	CTCCCTGGATCTTCCTTACA
Forward primer		
Reverse primer		

Mutant	AGAGAATAAACCTTCCCATGAACC	CTCCCTGGATCTTCCTTACG
Forward primer		
Reverse primer		

Common	AATGATGATCTGAATCAGGACTGT	CAGAAACTAATCAGTCAGAATTAAAGC
Reverse primer		
Forward primer		

Product size	206	154

TM:	57	56–57

**Table 2:** Background information of two groups of Iranian 18–24 years medical sciences students selected, caries free (*n* = 41) and those with DMFT ≥ 4 (*n* = 40).

Variables	Alternatives	Caries free *n* (%)	DMFT ≥ 4 *n* (%)
Gender	Male	24 (58.5)	17 (42.5)
	Female	17 (41.5)	23 (57.5)

Father's education	Academic	29 (70.7)	16 (40)
	Nonacademic	12 (29.3)	24 (60)

Mother's education	Academic	24 (58.5)	11 (27.5)
	Nonacademic	17 (41.5)	29 (70.7)

Tooth brushing habit	Recommended tooth brushing habit (RTH)^∗^	12 (29.3)	9 (22.5)
	Lack of RTH	29 (70.7)	31 (77.5)

Consumption of snacks containing sugar	Non-acceptable (once a day or more)	22 (53.7)	30 (75)
	Acceptable (less than once a day)	19 (46.3)	10 (25)

Dental check up	By him/herself or none	22 (53.7)	6 (15)
	By a dentist	19 (46.3)	34 (85)

Last dental check up	More than a year ago	23 (56.1)	12 (30)
	Within the last year	18 (43.9)	28 (70)

Dentist as parents	Yes	3 (7.3)	0
	No	38 (92.7)	40 (100)

The fluoride level of drinking water^∗∗^	Very low	29 (74.4)	13 (33.3)
	Low	10 (25.6)	26 (66.7)

AMELX gene genotypes^∗∗∗^	TT	33 (80.5)	33 (82.5)
	TC	6 (14.6)	6 (15)
	CC	1 (2.4)	1 (2.5)
	Not determined	1 (2.4)	0 (0)

ENAM gene genotypes	TT	34 (82.9)	39 (97.5)
	TC	6 (14.6)	1 (2.5)
	Not determined	1 (2.4)	0 (0)

^∗^Recommended tooth brushing habit (RTH) was defined as brushing more than once a day with regular or frequent use of fluoride toothpaste. ^∗∗^There were two missing values for this variable. ^∗∗∗^CC genotype was observed in only two of the study samples, which was excluded from analyses.

**Table 3:** Association of independent variables (including genotype of AMELX gene) with the caries status in the control group (caries free) compared to case group (DMFT ≥ 4).

	ES^∗^	SE^∗∗^	*P*	Odds ratio	Confidence interval 95%
Mother's academic education	−1.781	0.611	0.004	0.169	0.051–0.558
Dental check up by dentist	1.259	0.681	0.065	3.521	0.926–13.385
Last dental check up in last year	1.367	0.651	0.036	3.922	1.096–14.043
Low fluoride level of drinking water (compared to very low)	1.708	0.659	0.010	5.517	1.515–20.091

^∗^Estimate of strength. ^∗∗^Standard error.

**Table 4:** Association of independent variables (including genotype of ENAM gene) with the caries status in the control group (caries free) compared to case group (DMFT ≥ 4).

	ES^∗^	SE^∗∗^	*P*	Odds ratio	Confidence interval 95%
Female gender	1.389	0.697	0.046	4.010	1.022–15.733
Mother's academic education	-2.450	0.745	0.001	0.449	0.020–0.371
Eating sweet less than once a day	-1.125	0.675	0.096	4.969	0.087–1.219
Dental check up by dentist	1.603	0.716	0.025	4.616	1.221–20.220
Last dental check up in last year	1.112	0.688	0.106	3.042	0.789–11.723
Low fluoride level of drinking water (compared to very low)	1.825	0.705	0.010	6.201	1.556–24.711

^∗^Estimate of strength. ^∗∗^Standard error.

## Data Availability

The data used to support the findings of this study are included within the article.
